# Comparison of Different Parameters to Evaluate Delamination in Edge Trimming of Basalt Fiber Reinforced Plastics (BFRP)

**DOI:** 10.3390/ma13235326

**Published:** 2020-11-24

**Authors:** María Dolores Navarro-Mas, María Desamparados Meseguer, Joaquín Lluch-Cerezo, Juan Antonio García-Manrique

**Affiliations:** 1 Department of Mechanical Engineering and Materials, Universitat Politècnica de València, Camino de Vera s/n, 46022 Valencia, Spain; amesegue@mcm.upv.es (M.D.M.); jollucer@upvnet.upv.es (J.L.-C.); jugarcia@mcm.upv.es (J.A.G.-M.); 2 Engineering Research Team, Florida Universitària, 46470 Catarroja, Spain

**Keywords:** edge trimming, delamination measurement, basalt fiber reinforced plastic (BFRP)

## Abstract

Delamination is one of the main problems that occur when machining fiber-reinforced composite materials. In this work, Types I and II of delamination are studied separately in edge trimming of basalt fiber reinforced plastic (BFRP). For this purpose, one-dimensional and area delamination parameters are defined. One-dimensional parameters (Wa and Wb) allow to know average fibers length while the analysis of area delamination parameters (Sd) allow to evaluate delamination density. To study delamination, different tests are carried out modifying cutting parameters (cutting speed, feed per tooth and depth of cut) and material characteristics (fiber volume fraction and fiber orientation). Laminates with a lower fiber volume fraction do not present delamination. Attending to one-dimensional parameters it can be concluded that Type II delamination is more important than Type I and that a high depth of cut generates higher values of delamination parameters. An analysis of variance (ANOVA) is performed to study area parameters. Although delamination has a random nature, for each depth of cut, more influence variables in area delamination are firstly, feed per tooth and secondly, cutting speed.

## 1. Introduction

In aeronautical, naval and automotive sectors, application of fiber-reinforced polymeric matrix composite materials is increasing, mainly due to their lightness, high mechanical resistance and resistance to corrosion and high temperatures. Manufacturing processes of fiber-reinforced composite materials allow to obtain parts with a geometry close to the desired one. After curing, machining processes are required to eliminate excess of material and to achieve the required shapes and tolerances. However, fiber-reinforced composite materials behave differently in machining than metals, due to their heterogeneous nature and anisotropy [1].

One of the main problems that appear when machining fiber-reinforced composite materials is delamination. Delamination consists of a separation of the fabric layers from the composite material due to the force exerted by the cutting tool during machining process. This defect affects both the dimensional accuracy and the subsequent joining between parts [2]. It can cause reprocess the parts, with the loss of time that this entails, and even to discard the part. Delamination can be classified into three types: I, II and III [3]. Type I appears when the fibers are bent into the machining line, causing surface damage to the part. Type II appears when there are fibers that protrude from the machined edge, while Type III appears when there are fibers parallel to the machined edge. It can also appear simultaneously delaminates of Type I/II.

In the literature, the most studied factors to evaluate delamination produced after milling are cutting parameters, fiber orientation angle and cutting tool characteristics (material, teeth number, etc.) [4,5,6,7,8]. Cutting speed and feed are mainly studied [9,10,11,12], but only a few authors also include depth of cut [13] since most of them think that its influence on delamination is not as relevant as cutting speed and feed. Fiber orientation angle respect to machining direction is as well evaluated, with the aim of determining orientations to avoid or decrease delamination [14,15,16].

In milling process, delamination has been mainly studied in grooving and in edge trimming operations. In grooving, a “delamination factor” is defined, similar to the one used in drilling. Instead of referencing the delamination measurement to the hole diameter, reference is made to the groove width [17]. As in drilling, this measurement parameter is one-dimensional and only take into account Type I delamination, which causes damage to the part. This parameter does not evaluate other types of delamination, which protrude into the groove, and that could be more important than Type I delamination.

To evaluate delamination in milling, in both grooving [18] and edge trimming [19], different types of delamination are identified (Types I, II and III), determining the influence of each one and quantifying their values. In edge trimming, for each type of delamination, fibers length is measured, as well as their frequency of appearance, obtaining the average of the values. In addition to measure the length of the fibers that cause different types of delamination, areas occupied by these fibers can also be evaluated. In this way, in wood machining, there are studies that define a delamination factor as the relationship between the delamination area and the evaluated length [20]. This factor can also be applied to fiber-reinforced polymeric matrix composites. Another work that deals with the measurement of delaminated areas defines an area delamination factor as the ratio between the delaminated area and the initial area [21]. An image comparison method is used to determine the delaminated area, but no distinction between different types of delamination is made.

Generally, delamination occurs mostly in one of the layers of the part, the upper or the lower layer, and most factors defined above only evaluate this effect in one of the layers. However, delamination factors have also been defined to jointly evaluate this defect in both layers [22]. In this proposal, delamination areas in both layers are measured in the machined length, taking images from the top, the bottom and the front of the part.

In this paper different parameters are defined to evaluate the two most important types of delamination, Type I and Type II, in edge trimming of BFRP. Basalt fiber is completely inert, non-toxic and good thermal and electrical insulator, and has properties that make it performs better than glass fiber and slightly worse than carbon fiber [23]. All parameters defined and evaluated in this work can also be applied to carbon or glass fiber reinforced plastics.

Different tests are carried modifying cutting parameters (cutting speed, feed per tooth and depth of cut) and material characteristics (fiber volume fraction and fiber orientation) at different cutting times to evaluate delamination.

Delamination parameters are classified in one-dimensional parameters and area parameters. One-dimensional parameters (Wa and Wb) allow to evaluate the length of the fibers that protrudes or goes inward from the trimming edge, considering free spaces between yarns, and without considering these free spaces. These parameters allow to quantify the delamination in all experiments at different cutting times. Anyway, area parameters are also needed to evaluate density delamination. All these parameters allow to study delamination, but considering that delamination has a random nature. Additionally, an ANOVA study has been performed to quantify the effect of cutting conditions and fiber orientation angle in area parameters.

## 2. Materials and Methods

### 2.1. Materials

**Figure 1:**
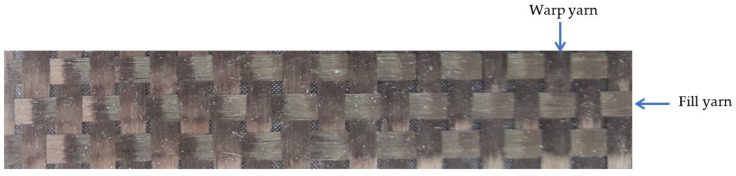
Bi-directional long basalt fiber fabric.

Parts to be machining are rectangular laminates (420 mm × 260 mm) of fiber-reinforced polymer matrix with a thickness of 3.4 mm manufactured by resin transfer molding (RTM). Reinforcement chosen is a bi-directional long basalt fiber fabric (Figure 1), with step between yarns 3 mm. Matrix is an epoxy low viscosity resin (Prime 20 LV Gurit, Newport Isle of Wight, UK).

Edge trimming operation (down milling) is conducted in a milling machine (Kondia B-500, Elgoibar, Spain), with a Mitsubishi Materials milling tool holder of diameter 25 mm and two exchangeable uncoated carbide cutting inserts [24]. To avoid dust, a film bag involved machining area is used.

Variables used to study delamination parameters are cutting parameters (cutting speed, feed per tooth and depth of cut) and material characteristics (fiber volume fraction and fiber orientation). Design of experiments is based in Taguchi’s method for five variables at two levels (Table 1). An L_16_ orthogonal array was selected to determine combination of factor levels to use for each experiment (Table 2). Each experiment consumes a large amount of material and an elevated time, therefore a full factorial design has not been considered, in order to reduce the number of experiments.

A cutting tool with new inserts was machining without coolant for 80 min. Stopping times (30, 50, 60, 70 and 80 min) are made to measure delamination parameters. In test 14, due to high cutting conditions, a great flank wear occurred, and it was not possible to reach a cutting time of 80 min.

### 2.2. Delamination Measurement Method

**Figure 2:**
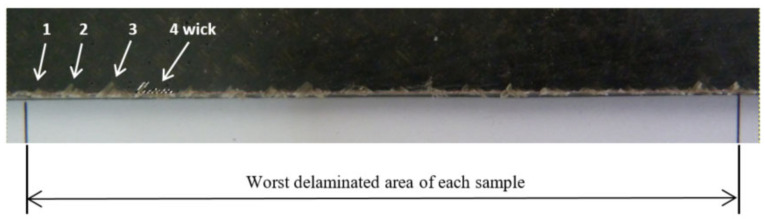
Evaluated laminate length (L).

Delamination measurement has been made capturing wick images of the upper face of laminates (Figure 2) in an evaluated length (L). Although laminates allow to measure 240 mm to evaluate delamination, only a length (L) of 80 mm is evaluated to reduce time and data acquisition cost. This length (L) is selected along of 240 mm of the laminate where delamination values are bigger.

**Figure 3:**
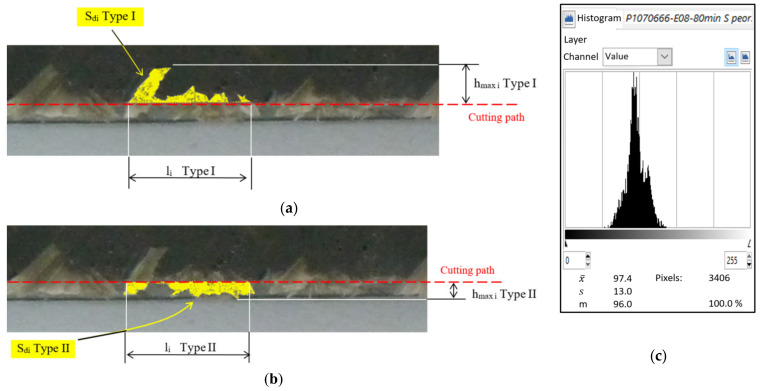
Example Wick number 4—parameters delamination measurement: (**a**) Type I delamination; (**b**) Type II delamination; (**c**) histogram with number of yellow pixels (Sdi, delaminated area).

An image is taken of the machined edge of the laminate, and for each wick (i) the following three parameters are captured by means of a cutout and its histogram: surface (Sdi) for each type of delamination; maximum value (hmax i) and invaded edge length (li) (Figure 3).

The values obtained by measuring wick to wick delamination from the captured images of each experiment are recorded in Excel data sheets, for subsequent analysis and calculation of delamination parameters. Table 3 shows data collected for test 12 at all cutting times, area delamination values (Sdi) and the length invaded (li) for Type II delamination on the trimming edge. All data are combined and summarized to obtain delamination parameters.

## 3. Results and Discussion

Laminates with a low fiber volume (Fv 40%) do not present delamination, so they will not be evaluated. However, in laminates manufactured with high fiber volume (Fv 60%) delamination always appears on the edge, regardless of the cutting time. In all laminates, delamination on the upper face is greater than on the lower face. In order to evaluate delamination only upper face will be measured.

**Figure 4:**
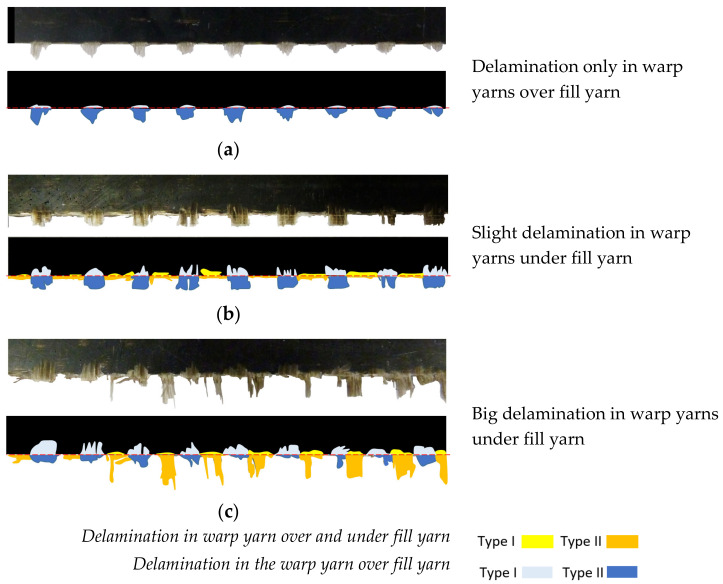
Delamination areas in 90° fiber orientation laminates. (**a**) Delamination in warp yarns over fill yarn (**b**,**c**) Delamination in warp yarns over and under the fill yarn.

Laminates with 45° and 90° fiber orientation are evaluated. In 45° fiber orientation laminates all warp yarns are over the fill yarn, thereby all wicks present delamination. In 90° fiber orientation, different delamination cases are studied (Figure 4), because delamination can appear only in the warp yarn over the fill yarn or in both, the warp yarns over and under the fill yarn.

**Figure 5:**
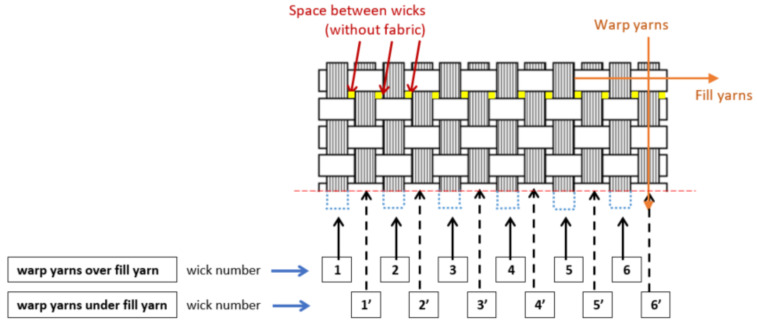
Warp yarns identification.

To analyze delamination, warp yarns over the fill yarn have been identified with a number. If warp yarns are under the fill yarn they have been identified with a number plus ‘(Figure 5).

In the evaluation of delamination, average length of the fibers that protrudes and goes inward from the laminate are measured. To quantify these values, one-dimensional parameters (Wa and Wb) are defined. Wa parameter allows to classify the tests attending at delamination magnitude, distributing delaminated surface in the evaluated length (L). Wb parameter calculates delamination magnitude, but not considering free space between wicks. On the other hand, in order to compare all experiments, a delamination factor (Fdel) is defined as the ratio between Wb value and the maximum theoretical possible value. This factor allows to classify experiment attending to the maximum possible theoretical delamination value. As this maximum value depends on the selected depth of cut, the effect of this variable is mitigated.

One-dimensional parameters do not define correctly delamination density. Therefore, it is also necessary to define area parameters (Sdi). As happens in one-dimensional parameters, to compare between experiments an area delamination factor (FSdi) is defined as the ratio between the Sdi and the maximum theoretical possible delaminated area for each experiment.

### 3.1. Length Delamination Parameters

These parameters allow to know the average length of fibers that protrude in Type II delamination and the average length of fibers that are inward from the trimmed edge in Type I delamination.

**Figure 6:**
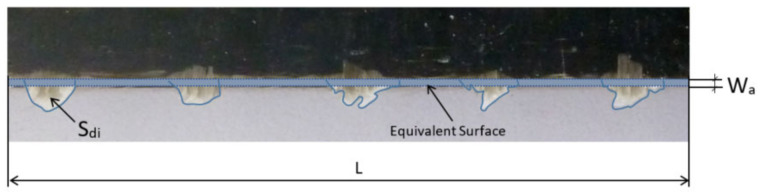
Medium delamination parameter (Wa) for Type II delamination.

The “medium delamination” parameter (Wa) is defined as the height of an equivalent rectangle of delaminated area with basis the evaluated length (L) (Figure 6). Studied length is 80 mm, and this length considers delaminated fibers and the free space between fibers, where there is not delamination:

(1) $$\mathrm{Wa}=\;\frac{\sum {\mathrm{Sd}}_{i}}{\;L\;}$$

**Figure 7:**
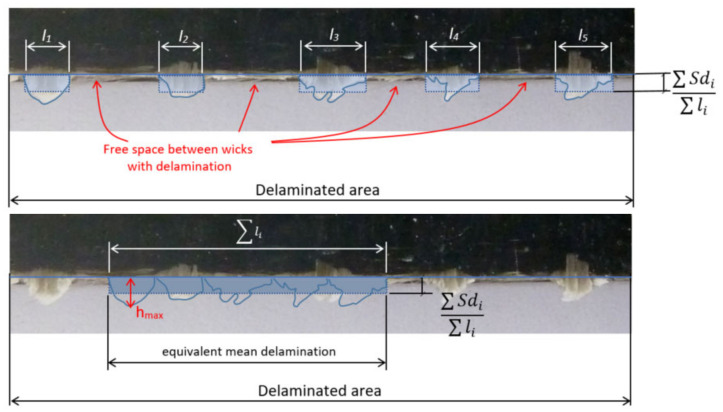
Equivalent delamination parameter (Wb) for Type II delamination.

Medium delamination parameter (Wa) does not allow a real measure of delamination length because all delaminated surface is related to studied length (L). Relating delaminated area to only delaminated fibers length ($\sum l_{i}),$ without considering free space between fibers, gives a more real approximation to average fibers length delamination. For this reason, a second parameter denominated “equivalent delamination” (Wb) is defined (Figure 7).

**Figure 8:**
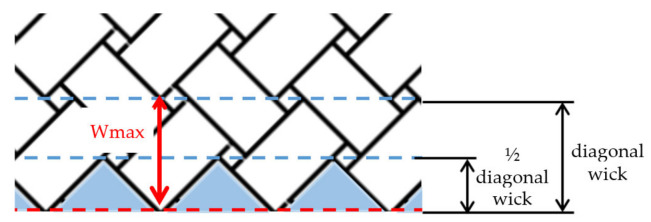
Theoretical maximum value of Wb in 45° fiber orientation tests.

In Type II delamination, the theoretical maximum value (Wmax) of parameters Wa and Wb is the depth of cut. In Type I delamination, this maximum value is the yarn measure (3 mm) in 90° fiber orientation tests, and the yarn diagonal (4.24 mm) in 45° fiber orientation tests (Figure 8). Delamination parameters are defined as mentioned above in warp yarns over fill yarn, and they are called Wa’ and Wb’ in warp yarns under fill yarn.

Table 4 and Table 5 show Wa, Wa’, Wb and Wb’ parameters and the theoretical maximum delamination (Wmax) values. To compare these values, delamination factors (Fdel and Fdel’) are defined as the ratio of Wb (or Wb’) respectively to Wmax:

(2) $$\mathrm{Fdel}=\;\frac{\mathrm{Wb}}{\;\mathrm{Wmax}\;}$$

(3) $$\mathrm{Fdel}\text{'}=\;\frac{\mathrm{Wb}\text{'}}{\;\mathrm{Wmax}\;}$$

**Figure 9:**
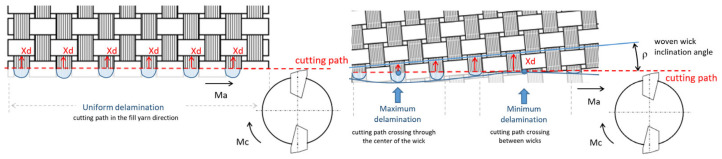
Xd variation.

In each experiment, data are taken at different cutting times. As cutting time increases, tool wear increases and delamination should be greater, increasing values of different parameters and factors. Delamination values present great variation at different cutting times, without an increasing or decreasing trend as tool wear increases. Table 4 and Table 5 show maximum and minimum delamination values for Wa, Wb and Fdel, observing that there is no correlation between delamination and cutting time. This is due to the fact that delamination has a random nature, mainly due to the distance of the warp yarn from the trimmed edge until the next dip below the crossing fill is different at every cutting time. This distance is defined as Xd in the literature [14]. Xd value depends on fiber orientation and the width of the yarn, as well as the inclination angle of the fabric with the cutting path. Xd can take a constant value, it can follow a uniform pattern or it can be totally random (Figure 9). For this reason, the minimum and maximum values of the analyzed delamination parameters and factors are not presented at the same cutting times, concluding that there is almost no dependence of delamination with cutting time.

In almost all experiments, Type I and Type II delamination appear at the same places of the trimming edge. Data from all experiments at all cutting times are analyzing, except for test 14, because in this test tool has reached a rapid flank wear and parameters values are excessive high. Values obtained for Type II delamination are much higher than values for Type I delamination. Table 6 shows average values for Wa, Wb and Fdel for Type I and II delamination and their comparison.

Depth of cut is one important parameter to evaluate delamination. Table 7 shows average delaminate values for parameters Wa, Wb and Fdel for experiments with different depth of cut and their comparison. A high depth of cut provokes a high delamination in the part (Table 7). This effect is not entirely showed in Fdel, because this factor is referenced to depth of cut.

In the test with 90° fiber orientation, Wa value is usually higher than Wa’. Table 4 and Table 5 show only a few cases where Wa and Wa’ present similar values. Therefore, it can be concluded that delamination of the warp yarn below the fill yarn is negligible. When parameters Wb and Wb’ are similar, delamination stands out the same, but delamination can be neglected as long as the length invaded is small. This means that there will only be a few wicks with delamination. If invaded length is not small, Wb and Wb’ should be considered together to evaluate delamination. In these tests, appearance of the machined piece is worst, with more length invaded by delamination.

**Figure 10:**
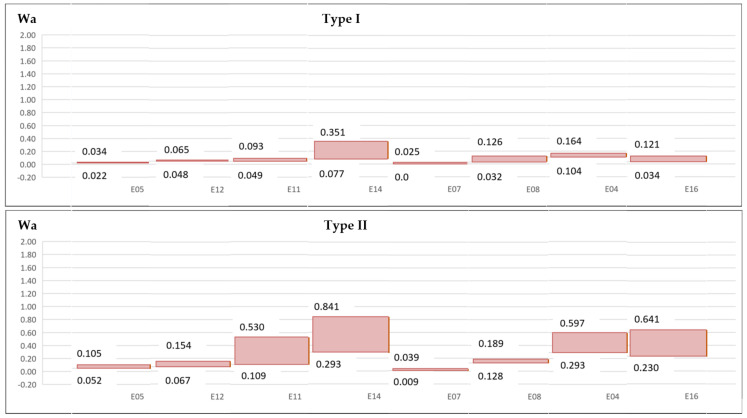

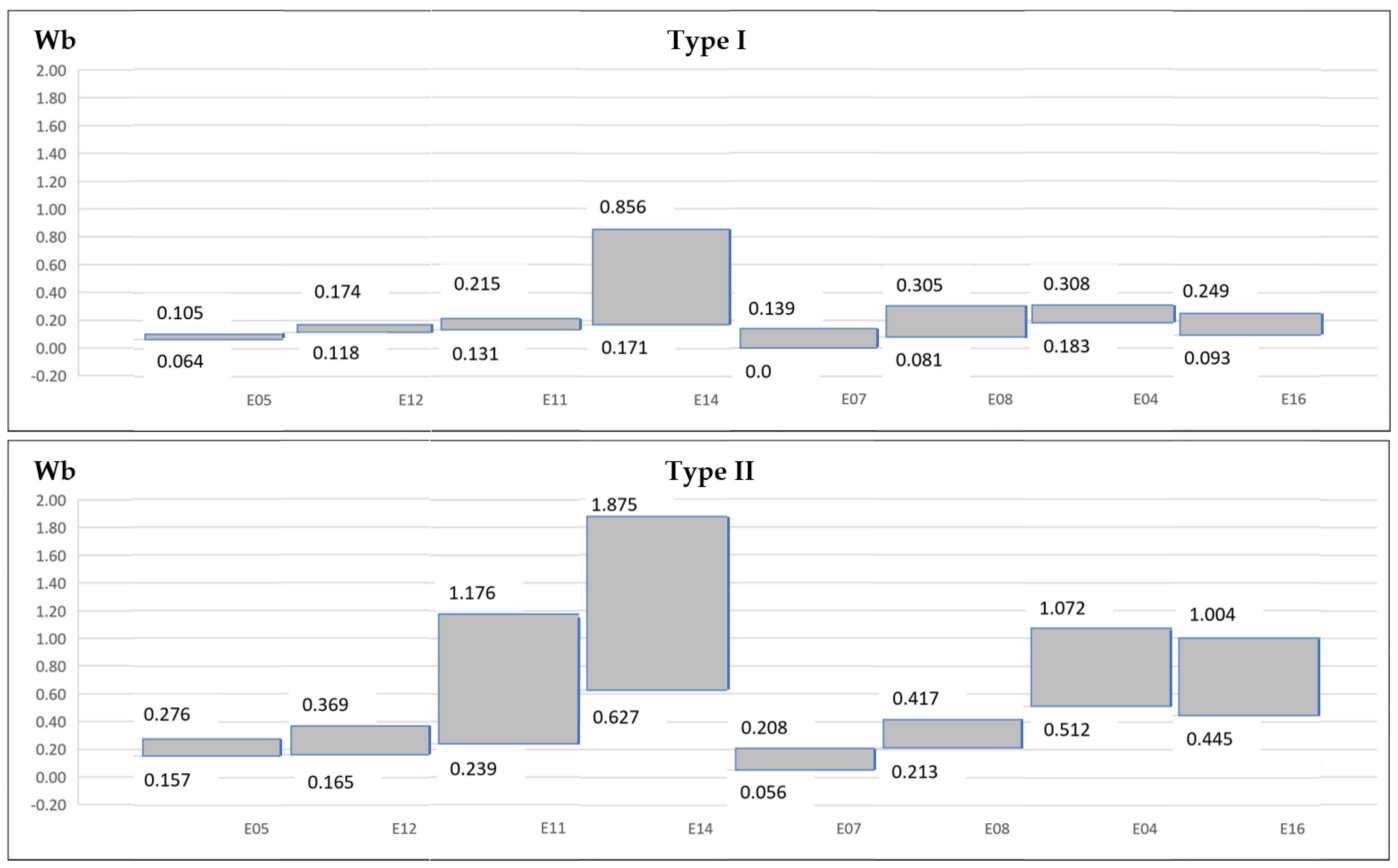
Wa and Wb delamination parameters.

**Figure 11:**
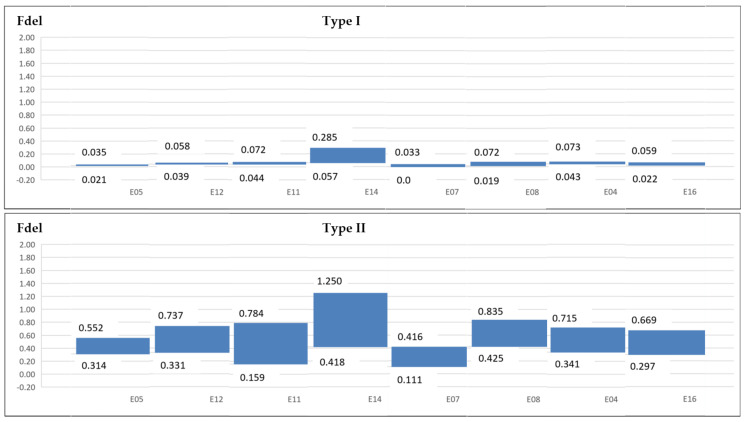
Fdel delamination factor.

Due to delamination values present great variation at different cutting times, without an increasing or decreasing trend, a range is established to delamination parameters and factors in each experiment (Figure 10 and Figure 11).

### 3.2. Area Delamination Parameters

In order to quantify density delamination in every experiment, values obtained for Wb and Wb’ parameters in 90° fiber orientation cannot be added. For this reason, delaminated areas measurement has been carried out. In 90° orientation fiber laminates, these areas are compared in the warp yarn over and under fill yarn.

As in one-dimensional parameters, area delamination factors (FSd) are also defined. In these parameters delaminated area is related to the maximum possible delamination area:

(4) $$\mathrm{FSdi}=\frac{\mathrm{Sdi}}{\;\mathrm{Sd}\;\max\;}$$

(5) $$\mathrm{FSdi}\text{'}=\frac{{\mathrm{Sdi}}^{\text{'}}}{\;\mathrm{Sd}\;\max\;}$$

Table 8 and Table 9 show these values and comparisons. Working with delamination areas allow to add FSdi and FSdi’ in 90° fiber orientation laminates to evaluate the total delaminated area, and comparing with 45° fiber orientation experiments.

**Figure 12:**
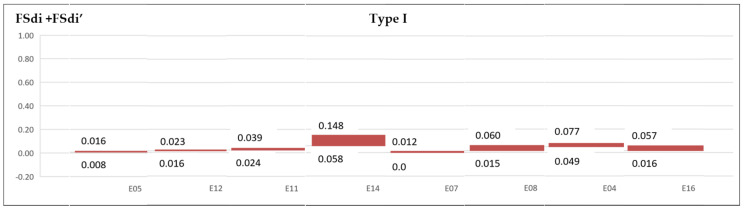
Area delamination factor range Type I (FSdi + FSdi’).

**Figure 13:**
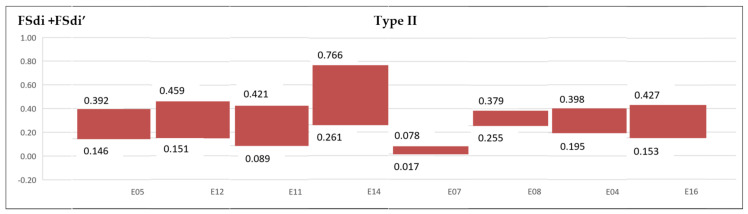
Area delamination factor range Type II (FSdi + FSdi’).

A range is established to delamination variation for FSdi and FSdi’ in each experiment for Type I and II delamination (Figure 12 and Figure 13).

An analysis of variance (ANOVA) has been performed to quantify the effect of cutting conditions and fiber orientation angle on the area delamination factors (FSdi + FSdi’). For analyzing the significant effect of the factors on the responses, F test with a level significance of 0.05 has been used. Table 10 shows that feed per tooth has the most significant effect on delamination, following by cutting speed. On the other hand fiber orientation is the least significant factor. It is expected that for a feed per tooth of 0.1 mm, delamination is smaller than for 0.4 mm. At smaller feeds, cutting edges impact on the fabric wick a greater number of times. Applying ANOVA to FSdi + FSdi’ does not allow to evaluate properly the effect of depth of cut, because this factor is referenced to depth of cut.

**Figure 14:**
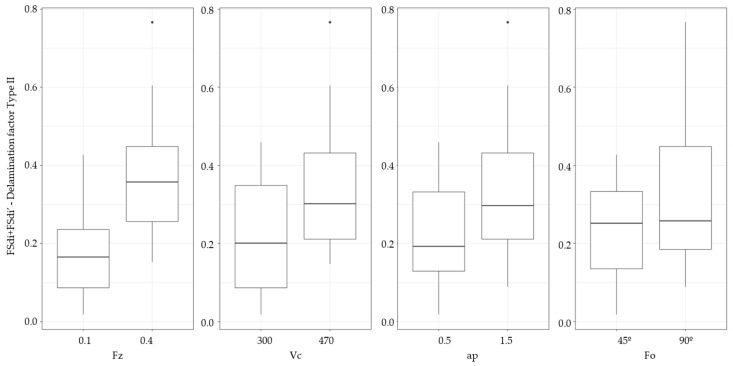
Box-whisker diagram.

The influence of each factor can be represented using a Box-whisker diagram (Figure 14). The mean plot shows the change in the response when variables varies from level 1 to level 2.

## 4. Conclusions

This paper defines and evaluates different delamination parameters for Type I and II delamination in edge trimming of basalt fiber reinforced plastics (BFRP). All parameters defined and evaluated in this work can also be applied to carbon or glass fiber reinforced plastics. For this purpose, different tests are carried out modifying cutting parameters (cutting speed, feed per tooth and depth of cut) and material characteristics (fiber volume fraction and fiber orientation). Delamination values have been obtained at different cutting times in order to find a relationship between delamination and cutting time, although most authors do not take delamination values at different cutting times.

Delamination parameters are classified in parameters that evaluate lengths and parameters that evaluate areas. Parameters that evaluate lengths give an average of protrude fibers length (for Type II delamination) and an average of inward fibers length (for Type I delamination). Two parameters are defined in this category (Wa and Wb). Wa considers free spaces between yarns, but Wb does not consider these free spaces. A delamination factor (Fdel) is also defined in order to compare delamination of each experiment with the maximum possible delamination value. On the other hand, parameters evaluating areas (Sd) are needed to quantify density delamination in the machining edge. Additionally, as in one-dimensional parameters, a factor (Fsd) is defined to compare area delamination.

Delamination at the top layer of the laminate only appears with a 60% fiber volume fraction. BFRP consists of a bi-directional long basalt fiber fabric, therefore delamination has been measured in warp yarns over and under fill yarn in 90° fiber orientation laminates. In most of 90° fiber orientation measurements, delamination measured in warp yarns over fill yarn is bigger than one measured in warp yarn under fill yarn, it can be concluded that delamination of the warp yarn below the fill yarn is negligible.

Delamination parameters and factors are calculated at different cutting times. Delamination values present great variation at different cutting times, without an increasing or decreasing trend as tool wear is increasing. It is due to the random nature of delamination. At every cutting time, the distance of the warp yarn from the trimmed edge until next dip below the crossing fill yarn (Xd) is changing, observing a uniform or sine wave delamination.

Analyzing one-dimensional parameters, it can be concluded that Type II delamination is more important than Type I and that a high depth of cut generates higher values of delamination parameters. Fdel factor can be used to compare between experiments with the same depth of cut, but not with different values of depth of cut, as maximum possible area depends on the selected depth of cut.

An analysis of variance (ANOVA) is performed to study area parameters. Although delamination has a random nature, for each depth of cut, more influence variables in area delamination are firstly, feed per tooth and secondly, cutting speed. It is expected that for a feed per tooth of 0.1 mm, delamination is smaller than for 0.4 mm, because at smaller feeds cutting edges impact on the fabric wick a greater number of times.

## Figures and Tables

**Table 1:** Variables and levels.

Level	Cutting Speed vc (m/Min)	Feed Per Tooth fz (mm)	Depth of Cut ap (mm)	Fiber Volume Fv (%)	Fiber Orientation Fo (°)
1	300	0.1	0.5	40	45
2	470	0.4	1.5	60	90

**Table 2:** Experimental parameters.

Test	Cutting Speed vc (m/Min)	Feed Per Tooth fz (mm)	Depth of Cut ap (mm)	Fiber Volume Fv (%)	Fiber Orientation Fo (°)
01	470	0.1	1.5	40	90
02	300	0.1	1.5	40	45
03	300	0.4	0.5	40	45
04	300	0.4	1.5	60	45
05	470	0.1	0.5	60	90
06	470	0.4	0.5	40	90
07	300	0.1	0.5	60	45
08	470	0.4	0.5	60	45
09	470	0.1	0.5	40	45
10	300	0.1	0.5	40	90
11	300	0.1	1.5	60	90
12	300	0.4	0.5	60	90
13	470	0.4	1.5	40	45
14	470	0.4	1.5	60	90
15	300	0.4	1.5	40	90
16	470	0.1	1.5	60	45

**Table 3:** Example of Type II delamination data sheets for test 12.

Tc	E12								Wicks								
(min)	Type II		1	2	3	4	5	6	7	8	9	10	11	12	13	14	Total
30	Sdi	(mm^2^)	1.12	0.86	1.06	1.05	1.29	0.90	1.31	1.22	0.93	0.54	0.57	0.80	0.45	0.22	12.33
	li	(mm)	2.00	2.13	3.48	2.57	3.16	2.93	2.82	2.51	2.53	1.96	1.71	2.17	2.80	0.72	33.47
50	Sdi	(mm^2^)	0.41	0.38	0.32	0.31	0.40	0.42	0.45	0.32	0.27	0.31	0.16	0.48	0.35	0.78	5.35
	li	(mm)	2.44	2.78	2.75	2.59	2.96	2.38	2.57	2.00	2.04	2.14	1.14	2.61	2.10	1.89	32.38
60	Sdi	(mm^2^)	0.41	0.38	0.32	0.31	0.40	0.42	0.45	0.32	0.27	0.31	0.16	0.48	0.35	0.78	5.35
	li	(mm)	2.42	2.75	2.73	2.57	2.94	2.35	2.54	1.98	2.02	2.12	1.11	2.59	2.08	1.87	32.08
70	Sdi	(mm^2^)	0.42	0.27	0.20	0.64	0.32	0.35	0.36	1.01	0.39	0.97	−	0.39	−	0.07	5.39
	li	(mm)	1.45	2.09	1.12	2.21	1.96	3.24	2.91	2.74	2.51	2.02	0	1.90	0	5.31	31.07
80	Sdi	(mm^2^)	0.87	0.90	0.88	0.90	0.72	0.60	0.64	0.53	0.61	0.66	0.36	0.37	0.48	0.48	9.03
	li	(mm)	2.60	2.20	1.99	2.18	2.09	2.43	2.03	1.73	2.51	2.77	2.03	2.15	2.32	2.72	31.74

**Table 4:** Parameter values in Type I delamination.

Test		Tc	Wa	Wa’	Wb	Wb’	Wmax	Fdel	Fdel’
		(min)	(mm)	(mm)	(mm)	(mm)	(mm)		
E05	Vc 470	30	0.022	0.003	0.069	0.051	3	0.0231	0.0171
	Fz 0.1	50	0.031	0.008	0.080	0.084	3	0.0265	0.0281
	ap 0.5	60	0.033	0.013	0.098	0.098	3	0.0326	0.0327
	Fo 90°	70	0.025	0.023	0.064	0.058	3	0.0215	0.0192
		80	0.034	0.012	0.105	0.051	3	0.0348	0.0169
E12	Vc 300	30	0.065	0.005	0.174	0.049	3	0.0579	0.0164
	Fz 0.4	50	0.048	0	0.118	0	3	0.0394	0
	ap 0.5	60	0.053	0	0.131	0	3	0.0437	0
	Fo 90°	70	0.062	0.004	0.160	0.091	3	0.0534	0.0303
		80	0.058	0.007	0.145	0.029	3	0.0485	0.0097
E11	Vc 300	30	0.081	0	0.178	0	3	0.0592	0
	Fz 0.1	50	0.093	0	0.215	0	3	0.0718	0
	ap 1.5	60	0.073	0.026	0.162	0.079	3	0.0539	0.0264
	Fo 90°	70	0.049	0.025	0.131	0.080	3	0.0438	0.0267
		80	0.063	0.054	0.133	0.102	3	0.0445	0.0340
E14	Vc 470	1.1	0.183	0.011	0.392	0.071	3	0.1308	0.0235
	Fz 0.4	5	0.147	0.124	0.309	0.272	3	0.1029	0.0905
	ap 1.5	10	0.222	0.154	0.498	0.278	3	0.1661	0.0928
	Fo 90°	20	0.351	0.094	0.852	0.161	3	0.2839	0.0535
		31	0.331	0.054	0.856	0.098	3	0.2852	0.0326
		37.5	0.161	0.012	0.430	0.070	3	0.1433	0.0232
		40	0.077	0.318	0.171	0.656	3	0.0571	0.2187
E07	Vc 300	30	0.007		0.139		4.24	0.0327	
	Fz 0.1	50	0.005		0.086		4.24	0.0202	
	ap 0.5	60	0.017		0.085		4.24	0.0201	
	Fo 45°	70	0		0		4.24	0	
		80	0.025		0.126		4.24	0.0296	
E08	Vc 470	30	0.126		0.266		4.24	0.0626	
	Fz 0.4	50	0.121		0.206		4.24	0.0486	
	ap 0.5	60	0.098		0.185		4.24	0.0436	
	Fo 45°	70	0.122		0.305		4.24	0.0719	
		80	0.032		0.081		4.24	0.0190	
E04	Vc 300	30	0.164		0.308		4.24	0.0726	
	Fz 0.4	50	0.105		0.225		4.24	0.0531	
	ap 1.5	60	0.137		0.238		4.24	0.0562	
	Fo 45°	70	0.104		0.183		4.24	0.0430	
		80	0.153		0.252		4.24	0.0593	
E16	Vc 470	30	0.039		0.098		4.24	0.0231	
	Fz 0.1	50	0.121		0.249		4.24	0.0587	
	ap 1.5	60	0.063		0.098		4.24	0.0231	
	Fo 45°	70	0.034		0.093		4.24	0.0219	
		80	0.086		0.180		4.24	0.0424	
maximum value 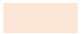			minimum value 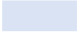				Wa≈Wa' 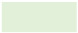		

**Table 5:** Parameter values in Type II delamination.

Test		Tc	Wa	Wa’	Wb	Wb’	Wmax	Fdel	Fdel’
		(min)	(mm)	(mm)	(mm)	(mm)	(mm)		
E05	Vc 470	30	0.065	0.015	0.201	0.170	0.5	0.4015	0.3404
	Fz 0.1	50	0.066	0.007	0.170	0.098	0.5	0.3402	0.1951
	ap 0.5	60	0.057	0.062	0.169	0.131	0.5	0.3386	0.2354
	Fo 90°	70	0.105	0.091	0.276	0.178	0.5	0.5522	0.3558
		80	0.052	0.062	0.157	0.164	0.5	0.3137	0.2730
E12	Vc 300	30	0.154	0.075	0.369	0.235	0.5	0.7371	0.4690
	Fz 0.4	50	0.067	0.022	0.165	0.136	0.5	0.3306	0.2722
	ap 0.5	60	0.067	0.009	0.167	0.127	0.5	0.3336	0.2549
	Fo 90°	70	0.067	0.010	0.173	0.175	0.5	0.3467	0.3498
		80	0.113	0.117	0.284	0.249	0.5	0.5688	0.4783
E11	Vc 300	30	0.109	0.025	0.239	0.093	1.5	0.1591	0.0618
	Fz 0.1	50	0.292	0.016	0.678	0.080	1.5	0.4520	0.0533
	ap 1.5	60	0.530	0.102	1.176	0.215	1.5	0.7839	0.1432
	Fo 90°	70	0.275	0.103	0.743	0.196	1.5	0.4952	0.1306
		80	0.276	0.070	0.581	0.133	1.5	0.3875	0.0886
E14	Vc 470	1.1	0.293	0.099	0.627	0.266	1.5	0.4180	0.1772
	Fz 0.4	5	0.643	0.179	1.352	0.341	1.5	0.9012	0.2270
	ap 1.5	10	0.538	0.368	1.207	0.663	1.5	0.8045	0.4422
	Fo 90°	20	0.464	0.201	1.125	0.342	1.5	0.7502	0.2282
		31	0.515	0.222	1.333	0.401	1.5	0.8884	0.2674
		37.5	0.484	0.166	1.293	0.362	1.5	0.8623	0.2410
		40	0.841	0.308	1.875	0.634	1.5	1.2502	0.4226
E07	Vc 300	30	0.028		0.208		0.5	0.4160	
	Fz 0.1	50	0.021		0.120		0.5	0.2400	
	ap 0.5	60	0.009		0.056		0.5	0.1112	
	Fo 45°	70	0.030		0.182		0.5	0.3646	
		80	0.039		0.149		0.5	0.2971	
E08	Vc 470	30	0.189		0.397		0.5	0.7932	
	Fz 0.4	50	0.165		0.283		0.5	0.5654	
	ap 0.5	60	0.136		0.256		0.5	0.5115	
	Fo 45°	70	0.168		0.417		0.5	0.8349	
		80	0.128		0.213		0.5	0.4250	
E04	Vc 300	30	0.372		0.745		1.5	0.4967	
	Fz 0.4	50	0.498		1.072		1.5	0.7148	
	ap 1.5	60	0.596		1.038		1.5	0.6921	
	Fo 45°	70	0.293		0.512		1.5	0.3412	
		80	0.597		0.983		1.5	0.6551	
E16	Vc 470	30	0.387		0.848		1.5	0.5652	
	Fz 0.1	50	0.248		0.513		1.5	0.3420	
	ap 1.5	60	0.641		1.004		1.5	0.6693	
	Fo 45°	70	0.230		0.683		1.5	0.4552	
		80	0.245		0.445		1.5	0.2969	
maximum value 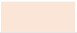			minimum value 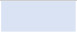				Wa≈Wa' 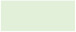		

**Table 6:** Type I and II delamination comparison.

	$\bar{W_{a}}$ (**mm**)	$\bar{W_{b}}\;\;\left(\mathrm{mm}\right)$	$\bar{F_{del}}$
Type I	0.067	0.153	0.042
Type II	0.209	0.448	0.467
			
	$\frac{\bar{W_{a}}\;\mathrm{Type}\;\mathrm{II}}{\bar{W_{a}}\;\mathrm{Type}\;I\;}=3.12$	$\frac{\bar{W_{b}}\;\mathrm{Type}\;\mathrm{II}}{\bar{W_{b}}\;\mathrm{Type}\;I\;}=2.93$	$\frac{\bar{F_{\mathrm{del}}}\;\mathrm{Type}\;\mathrm{II}}{\bar{F_{\mathrm{del}}}\;\mathrm{Type}\;I\;}=11.12$

**Table 7:** Comparison of experiments with different depth of cut.

**ap (mm)**	$\bar{W_{a}}$ **(mm)**	$\bar{W_{b}}\;\;\left(mm\right)$	$\bar{F_{del}}$
0.5	0.086	0.221	0.441
1.5	0.373	0.751	0.500
			
	$\frac{\bar{W_{a}}\;\mathrm{ap}1.5}{\bar{W_{a}}\;\mathrm{ap}0.5}=4.32$	$\frac{\bar{W_{b}}\;\mathrm{ap}1.5}{\bar{W_{b}}\;\mathrm{ap}0.5}=3.40$	$\frac{\bar{F_{\mathrm{del}}}\;\mathrm{ap}1.5}{\bar{F_{\mathrm{del}}}\;\mathrm{ap}0.5}=1.13$

**Table 8:** Area parameters in Type I delamination.

Test		Tc	Sdi	Sdi‘	Sdmax	FSdi	FSdi’	FSdi + FSdi’
		(min)	(mm^2^)	(mm^2^)	(mm^2^)			
E05	Vc 470	30	1.79	0.21	240	0.0075	0.0009	0.0084
	Fz 0.1	50	2.48	0.63	240	0.0103	0.0026	0.0130
	ap 0.5	60	2.64	1.01	240	0.0110	0.0042	0.0152
	Fo 90°	70	1.97	1.83	240	0.0082	0.0076	0.0158
		80	2.75	0.94	240	0.0114	0.0039	0.0153
E12	Vc 300	30	5.20	0.37	240	0.0217	0.0015	0.0232
	Fz 0.4	50	3.82	0	240	0.0159	0	0.0159
	ap 0.5	60	4.21	0	240	0.0175	0	0.0175
	Fo 90°	70	4.98	0.31	240	0.0207	0.0013	0.0220
		80	4.61	0.54	240	0.0192	0.0022	0.0215
E11	Vc 300	30	6.46	0	240	0.0269	0	0.0269
	Fz 0.1	50	7.42	0	240	0.0309	0	0.0309
	ap 1.5	60	5.83	2.10	240	0.0243	0.0088	0.0331
	Fo 90°	70	3.89	1.96	240	0.0162	0.0082	0.0244
		80	5.07	4.29	240	0.0211	0.0179	0.0390
E14	Vc 470	1.1	14.65	0.84	240	0.0611	0.0035	0.0646
	Fz 0.4	5	11.74	9.94	240	0.0489	0.0414	0.0903
	ap 1.5	10	17.77	12.34	240	0.0740	0.0514	0.1254
	Fo 90°	20	28.08	7.55	240	0.1170	0.0315	0.1485
		31	26.46	4.33	240	0.1102	0.0180	0.1283
		37.5	12.87	0.94	240	0.0536	0.0039	0.0575
		40	6.15	25.46	240	0.0256	0.1061	0.1317
E07	Vc 300	30	0.59		169.71	0.0035		0.0035
	Fz 0.1	50	0.43		169.71	0.0025		0.0025
	ap 0.5	60	1.35		169.71	0.0079		0.0079
	Fo 45°	70	0		169.71	0		0
		80	2.01		169.71	0.0118		0.0118
E08	Vc 470	30	10.1		169.71	0.0595		0.0595
	Fz 0.4	50	9.65		169.71	0.0569		0.0569
	ap 0.5	60	7.86		169.71	0.0463		0.0463
	Fo 45°	70	9.79		169.71	0.0577		0.0577
		80	2.58		169.71	0.0152		0.0152
E04	Vc 300	30	13.09		169.71	0.0772		0.0772
	Fz 0.4	50	8.37		169.71	0.0493		0.0493
	ap 1.5	60	10.96		169.71	0.0646		0.0646
	Fo 45°	70	8.35		169.71	0.0492		0.0492
		80	12.23		169.71	0.0721		0.0721
E16	Vc 470	30	3.12		169.71	0.0184		0.0184
	Fz 0.1	50	9.64		169.71	0.0568		0.0568
	ap 1.5	60	5.02		169.71	0.0296		0.0296
	Fo 45°	70	2.75		169.71	0.0162		0.0162
		80	6.85		169.71	0.0403		0.0403

**Table 9:** Area parameters in Type II delamination.

Test		Tc	Sdi	Sdi‘	Sdmax	FSdi	FSdi’	FSdi + FSdi’
		(min)	(mm^2^)	(mm^2^)	(mm^2^)			
E05	Vc 470	30	5.19	1.17	40	0.1297	0.0292	0.1588
	Fz 0.1	50	5.30	0.56	40	0.1324	0.0139	0.1464
	ap 0.5	60	4.56	4.99	40	0.1141	0.1125	0.2266
	Fo 90°	70	8.42	7.27	40	0.2105	0.1818	0.3922
		80	4.12	4.94	40	0.1030	0.1025	0.2055
E12	Vc 300	30	12.33	6.03	40	0.3084	0.1507	0.4590
	Fz 0.4	50	5.35	1.76	40	0.1338	0.0441	0.1779
	ap 0.5	60	5.35	0.70	40	0.1338	0.0175	0.1513
	Fo 90°	70	5.39	0.79	40	0.1346	0.0198	0.1544
		80	9.03	9.37	40	0.2257	0.2250	0.4507
E11	Vc 300	30	8.68	1.96	120	0.0724	0.0163	0.0887
	Fz 0.1	50	23.35	1.24	120	0.1946	0.0104	0.2050
	ap 1.5	60	42.40	8.18	120	0.3533	0.0682	0.4215
	Fo 90°	70	21.99	8.27	120	0.1832	0.0690	0.2522
		80	22.08	5.58	120	0.1840	0.0465	0.2305
E14	Vc 470	1.1	23.41	7.95	120	0.1951	0.0662	0.2614
	Fz 0.4	5	51.42	14.29	120	0.4285	0.1191	0.5476
	ap 1.5	10	43.04	29.41	120	0.3586	0.2451	0.6037
	Fo 90°	20	37.10	16.10	120	0.3091	0.1341	0.4433
		31	41.21	17.77	120	0.3434	0.1481	0.4915
		37.5	38.73	13.28	120	0.3228	0.1106	0.4334
		40	67.29	24.60	120	0.5608	0.2050	0.7658
E07	Vc 300	30	2.23		40	0.0558		0.0558
	Fz 0.1	50	1.68		40	0.0420		0.0420
	ap 0.5	60	0.68		40	0.0171		0.0171
	Fo 45°	70	2.37		40	0.0593		0.0593
		80	3.12		40	0.0780		0.0780
E08	Vc 470	30	15.15		40	0.3788		0.3788
	Fz 0.4	50	13.23		40	0.3308		0.3308
	ap 0.5	60	10.87		40	0.2718		0.2718
	Fo 45°	70	13.4		40	0.3350		0.3350
		80	10.2		40	0.2550		0.2550
E04	Vc 300	30	29.77		120	0.2481		0.2481
	Fz 0.4	50	39.85		120	0.3320		0.3320
	ap 1.5	60	47.71		120	0.3976		0.3976
	Fo 45°	70	23.42		120	0.1951		0.1951
		80	47.73		120	0.3978		0.3978
E16	Vc 470	30	30.93		120	0.2578		0.2578
	Fz 0.1	50	19.87		120	0.1656		0.1656
	ap 1.5	60	51.29		120	0.4274		0.4274
	Fo 45°	70	18.41		120	0.1534		0.1534
		80	19.57		120	0.1631		0.1631

**Table 10:** Analysis of variance (ANOVA).

Factor	Df	Sum Sq	Mean Sq	F Value	Pr (>F)
Fz	1	0.3395	0.3395	27.876	5.93 × 10^6^
Vc	1	0.1228	0.1228	10.084	0.00301
ap	1	0.1271	0.1271	10.432	0.00260
Fo	1	0.0758	0.0758	6.221	0.01722
